# Regarding: ‘qualitative analysis of out-of-hospital self-management capabilities and ongoing care needs in patients with gynecological malignancies and venous thromboembolism’

**DOI:** 10.1080/07853890.2026.2638087

**Published:** 2026-02-27

**Authors:** Yifan Ye

**Affiliations:** Medical Engineering Department, The First Affiliated Hospital of Hebei North University, Zhangjiakou, Hebei Province, China

To the Editor

I read with great interest the study by Li et al. regarding the self-management of venous thromboembolism (VTE) in patients with gynecological malignancies [1]. The manuscript effectively captures the ‘low self-efficacy’ and ‘high family dependence’ characteristic of this population. To further elevate the study’s clinical applicability and alignment with precision nursing, I offer the following forward-looking recommendations for your consideration.

From a clinical data perspective, it would be valuable to more clearly distinguish the physiological drivers of immobility. While the current analysis attributes low physical activity primarily to motivation or surgical site pain, the fact that participants were readmitted for chemotherapy suggests a need to stratify mechanical surgical limitations from chemotherapy-related fatigue (CRF). In predictive modeling, these represent distinct physiological states requiring different interventions – analgesia for the former versus energy conservation techniques for the latter – and differentiating them would significantly refine the ‘regulatory’ dimension of your self-care model [2].

Beyond physical constraints, the finding that patients lack interest in VTE information may reflect a competing risk prioritization rather than a simple knowledge deficit. For patients facing advanced malignancies, such as the Stage III and IV ovarian cancer cases in your cohort, the immediate existential threat of tumor progression often naturally overshadows potential complications like VTE [3]. Future inquiries could assess the ‘concern ratio’ between cancer survival and thrombosis; if patients view VTE prevention as a distraction from their primary battle, standard education will remain ineffective regardless of the delivery method.

When considering educational formats, the suggestion to use leaflets for elderly or illiterate patients might benefit from a closer examination of the digital phenotypes in your data. For instance, Patient A06 explicitly noted being text-illiterate yet capable of navigating mobile video platforms. This discrepancy suggests that ‘illiteracy’ is not a barrier to digital health, but rather an invitation to explore Voice-UI agents or Generative AI video avatars. Aligning interventions with these observed behaviors offers a more scalable and measurable solution than static print materials [4].

Finally, to truly bridge the gap between hospital and home, the field is moving beyond reliance on subjective symptom recognition – which you noted is often poor – toward medical-engineering convergence. The integration of wearable biosensors [5] to objectively monitor limb circumference and skin temperature represents the next frontier in VTE prevention. Implementing such systems requires cultivating interdisciplinary ‘nurse-engineers’ capable of managing remote digital ecosystems. Positioning your findings within this trajectory would greatly enhance the study’s relevance to future care models.

## Data Availability

There is no data associated with this research.
